# Di-μ-chlorido-bis­[(2,2′-bibenzimidazole)chloridocadmium(II)]

**DOI:** 10.1107/S1600536808040968

**Published:** 2008-12-10

**Authors:** Ge Liu

**Affiliations:** a Chifeng University, Chifeng 024000, People’s Republic of China

## Abstract

The title binuclear complex, [Cd_2_Cl_4_(C_14_H_10_N_4_)_2_], was synthesized by the hydro­thermal reaction of CdCl_2_ and the ligand 2,2′-bibenzimidazole. The mol­ecule lies on an inversion center and the metal center displays a strongly distorted trigonal-bipyramidal geometry. The Cd^II^ ions are coordinated by two N atoms from the organic ligand, and by one terminal and two bridging chloride anions. The crystal structure involves inter­molecular N—H⋯Cl hydrogen bonds, resulting in a one-dimensional supra­molecular structure.

## Related literature

For the synthesis of 2,2′-bibenzimidazole, see: Fieselmann *et al.* (1978 ▶). For general properties of Cd^II^-based complex polymers, see: Meng *et al.* (2004 ▶).
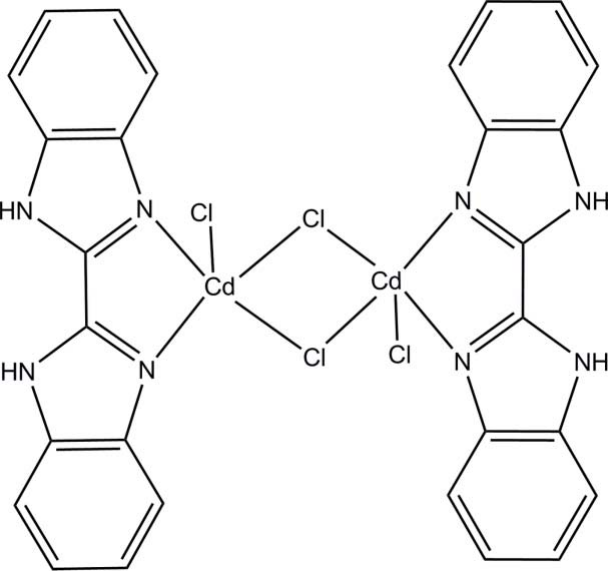


## Experimental

### 

#### Crystal data

[Cd_2_Cl_4_(C_14_H_10_N_4_)_2_]
*M* *_r_* = 835.12
Monoclinic, 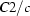

*a* = 11.824 (2) Å
*b* = 10.784 (2) Å
*c* = 22.828 (5) Å
β = 91.10 (3)°
*V* = 2910.1 (10) Å^3^
*Z* = 4
Mo *K*α radiation
μ = 1.86 mm^−1^
*T* = 293 (2) K
0.17 × 0.16 × 0.12 mm

#### Data collection

Rigaku R-AXIS RAPID-S diffractometer
Absorption correction: none
14677 measured reflections
3337 independent reflections
2840 reflections with *I* > 2σ(*I*)
*R*_int_ = 0.035

#### Refinement

*R*[*F* ^2^ > 2σ(*F* ^2^)] = 0.033
*wR*(*F* ^2^) = 0.060
*S* = 1.14
3337 reflections
190 parameters
H-atom parameters constrained
Δρ_max_ = 0.29 e Å^−3^
Δρ_min_ = −0.34 e Å^−3^

### 

Data collection: *RAPID-AUTO* (Rigaku, 1998 ▶); cell refinement: *RAPID-AUTO*; data reduction: *CrystalStructure* (Rigaku/MSC, 2002 ▶); program(s) used to solve structure: *SHELXS97* (Sheldrick, 2008 ▶); program(s) used to refine structure: *SHELXL97* (Sheldrick, 2008 ▶); molecular graphics: *SHELXTL* (Sheldrick, 2008 ▶); software used to prepare material for publication: *SHELXTL*.

## Supplementary Material

Crystal structure: contains datablocks I, global. DOI: 10.1107/S1600536808040968/bh2211sup1.cif

Structure factors: contains datablocks I. DOI: 10.1107/S1600536808040968/bh2211Isup2.hkl

Additional supplementary materials:  crystallographic information; 3D view; checkCIF report

## Figures and Tables

**Table:** 

Cd1—N4	2.305 (2)
Cd1—N1	2.338 (2)
Cd1—Cl2	2.4602 (8)
Cd1—Cl1	2.5725 (10)
Cd1—Cl1^i^	2.5903 (10)

**Table:** 

N4—Cd1—Cl2	118.63 (6)
N4—Cd1—Cl1	144.04 (6)
Cl2—Cd1—Cl1	96.65 (3)
N1—Cd1—Cl1^i^	154.49 (6)

Symmetry code: (i) 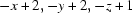.

**Table 2:** Hydrogen-bond geometry (Å, °)

*D*—H⋯*A*	*D*—H	H⋯*A*	*D*⋯*A*	*D*—H⋯*A*
N3—H16⋯Cl2^ii^	0.86	2.39	3.221 (2)	163

Symmetry code: (ii) 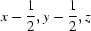.

**Fig. 1.**
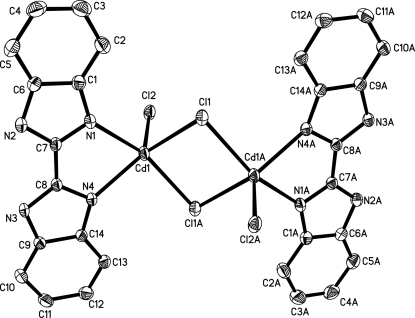
The structure of the title compound with displacement ellipsoids at the 30% probability level.

**Fig. 2.**
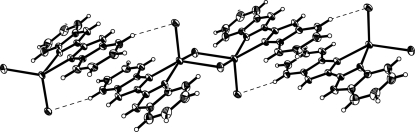
One dimensional chain formed by hydrogen bonds (dashed lines) in the crystal structure of the title compound.

## supplementary crystallographic information

### Comment

Bibenzimidazole has the potential to function as a bis-bidentate nitrogen ligand by coordinating to metal ions as a chelate. On the other hand, Cd^II^-containing coordination polymers have attracted much attention as they are able to bond to different donors ligands simultaneously, because of the Cd^II^ large radius. Various coordination modes and potential applications in catalysis, fluorescent materials, NLO materials and so on (Meng *et al.* 2004) have been described. Here we report the crystal structure of the title complex prepared from CdCl_2_ and bibenzimidazole ligand (see experimental).

As show in Fig. 1, the complex lies on an inversion center, and Cd atoms have strongly distorted trigonal-bipyramidal geometry, being coordinated by two N atoms from the organic ligand, and by one terminal and two bridging Cl^-^ anions. The two Cd centers are bridged by two chloride ions to give a dinuclear cadmium complex. Intermolecular N—H···Cl hydrogen bonds extend the dinuclear complex to a one dimensional chain in the crystal structure (Fig. 2).

### Experimental

A mixture of CdCl_2_ (0.073 g, 0.40 mmol), bibenzimidazole (0.070 g, 0.30 mmol) and H_2_O (10 ml) was placed in a Teflon reactor, then heated to 433 K at 10.8 K/h; after maintaining the reaction at 433 K for three days, it was cooled to 303 K at 10.8 K/h. Crystals suitable for X-ray analysis were obtained.

### Refinement

Raw diffraction data were used for refinement, since semi-empirical correction failed to properly correct absorption effects. All H atoms were placed in geometrically idealized positions and constrained to ride on their parent atoms, with N—H = 0.86 Å, C—H = 0.93 Å, and with *U*_iso_(H) = 1.2*U*_eq_(carrier atom).

### Crystal data

**Table:**

[Cd_2_Cl_4_(C_14_H_10_N_4_)_2_]	*F*(000) = 1632
*M**_r_* = 835.12	*D*_x_ = 1.906 Mg m^−^^3^
Monoclinic, *C*2/*c*	Mo *K*α radiation, λ = 0.71073 Å
Hall symbol: -C 2yc	Cell parameters from 13595 reflections
*a* = 11.824 (2) Å	θ = 3.1–27.5°
*b* = 10.784 (2) Å	µ = 1.86 mm^−^^1^
*c* = 22.828 (5) Å	*T* = 293 K
β = 91.10 (3)°	Prism, yellow
*V* = 2910.1 (10) Å^3^	0.17 × 0.16 × 0.12 mm
*Z* = 4	

### Data collection

**Table:**

Rigaku R-AXIS RAPID-S diffractometer	2840 reflections with *I* > 2σ(*I*)
Radiation source: fine-focus sealed tube	*R*_int_ = 0.035
graphite	θ_max_ = 27.5°, θ_min_ = 3.1°
ω scans	*h* = −15→15
14677 measured reflections	*k* = −14→14
3337 independent reflections	*l* = −29→29

### Refinement

**Table:**

Refinement on *F*^2^	Primary atom site location: structure-invariant direct methods
Least-squares matrix: full	Secondary atom site location: difference Fourier map
*R*[*F*^2^ > 2σ(*F*^2^)] = 0.033	Hydrogen site location: inferred from neighbouring sites
*wR*(*F*^2^) = 0.060	H-atom parameters constrained
*S* = 1.14	*w* = 1/[σ^2^(*F*_o_^2^) + (0.0213*P*)^2^ + 2.9529*P*] where *P* = (*F*_o_^2^ + 2*F*_c_^2^)/3
3337 reflections	(Δ/σ)_max_ = 0.002
190 parameters	Δρ_max_ = 0.29 e Å^−^^3^
0 restraints	Δρ_min_ = −0.34 e Å^−^^3^

### Fractional atomic coordinates and isotropic or equivalent isotropic displacement parameters (Å^2^)

**Table:**

	*x*	*y*	*z*	*U*_iso_*/*U*_eq_	
C1	0.7642 (2)	0.8420 (3)	0.65287 (12)	0.0352 (6)	
C2	0.8455 (3)	0.8628 (3)	0.69666 (14)	0.0488 (8)	
H2	0.9096	0.9102	0.6896	0.059*	
C3	0.8279 (3)	0.8110 (3)	0.75062 (15)	0.0567 (9)	
H3	0.8804	0.8252	0.7807	0.068*	
C4	0.7338 (3)	0.7380 (3)	0.76151 (14)	0.0569 (9)	
H4	0.7252	0.7037	0.7985	0.068*	
C5	0.6533 (3)	0.7156 (3)	0.71877 (13)	0.0523 (9)	
H5	0.5909	0.6658	0.7259	0.063*	
C6	0.6688 (2)	0.7699 (3)	0.66449 (12)	0.0370 (6)	
C7	0.6614 (2)	0.8380 (2)	0.57409 (11)	0.0314 (6)	
C8	0.6263 (2)	0.8657 (2)	0.51432 (11)	0.0314 (6)	
C9	0.5301 (2)	0.8803 (2)	0.43105 (11)	0.0319 (6)	
C10	0.4514 (2)	0.8770 (3)	0.38526 (13)	0.0409 (7)	
H10	0.3817	0.8381	0.3889	0.049*	
C11	0.4822 (3)	0.9342 (3)	0.33429 (13)	0.0450 (7)	
H11	0.4318	0.9343	0.3025	0.054*	
C12	0.5875 (3)	0.9926 (3)	0.32871 (13)	0.0441 (7)	
H12	0.6050	1.0302	0.2934	0.053*	
C13	0.6652 (2)	0.9957 (2)	0.37397 (12)	0.0378 (6)	
H13	0.7349	1.0343	0.3700	0.045*	
C14	0.6356 (2)	0.9386 (2)	0.42626 (11)	0.0306 (6)	
Cd1	0.851704 (16)	1.006362 (18)	0.527622 (9)	0.03487 (7)	
Cl1	1.04814 (6)	0.95042 (8)	0.57022 (3)	0.04315 (18)	
Cl2	0.84050 (6)	1.21444 (7)	0.57132 (3)	0.04270 (18)	
N1	0.75762 (18)	0.8824 (2)	0.59515 (10)	0.0343 (5)	
N2	0.60522 (19)	0.7697 (2)	0.61309 (9)	0.0386 (6)	
H15	0.5416	0.7327	0.6072	0.046*	
N3	0.52742 (18)	0.8349 (2)	0.48775 (9)	0.0347 (5)	
H16	0.4728	0.7946	0.5032	0.042*	
N4	0.69432 (18)	0.9280 (2)	0.47931 (9)	0.0324 (5)	

### Atomic displacement parameters (Å^2^)

**Table:**

	*U*^11^	*U*^22^	*U*^33^	*U*^12^	*U*^13^	*U*^23^
C1	0.0327 (15)	0.0355 (15)	0.0373 (15)	0.0013 (11)	0.0013 (12)	0.0007 (12)
C2	0.0377 (17)	0.058 (2)	0.0510 (19)	−0.0033 (15)	−0.0065 (15)	0.0030 (16)
C3	0.051 (2)	0.071 (2)	0.047 (2)	0.0076 (18)	−0.0105 (16)	−0.0022 (18)
C4	0.058 (2)	0.077 (3)	0.0352 (18)	0.0122 (19)	0.0038 (16)	0.0087 (17)
C5	0.0448 (19)	0.067 (2)	0.0455 (19)	−0.0043 (16)	0.0112 (15)	0.0063 (16)
C6	0.0329 (15)	0.0432 (17)	0.0353 (15)	0.0006 (12)	0.0052 (12)	−0.0024 (13)
C7	0.0283 (14)	0.0314 (14)	0.0348 (14)	−0.0073 (11)	0.0049 (11)	−0.0026 (11)
C8	0.0276 (14)	0.0320 (14)	0.0348 (14)	−0.0081 (11)	0.0037 (11)	−0.0045 (11)
C9	0.0307 (14)	0.0312 (14)	0.0338 (14)	−0.0053 (11)	0.0019 (11)	−0.0052 (11)
C10	0.0329 (15)	0.0450 (17)	0.0446 (17)	−0.0079 (13)	−0.0029 (13)	−0.0052 (14)
C11	0.0461 (18)	0.0484 (18)	0.0400 (17)	−0.0064 (14)	−0.0103 (14)	−0.0030 (14)
C12	0.0552 (19)	0.0410 (17)	0.0363 (15)	−0.0103 (14)	0.0022 (14)	0.0006 (13)
C13	0.0383 (15)	0.0364 (15)	0.0389 (15)	−0.0119 (12)	0.0071 (12)	−0.0028 (13)
C14	0.0302 (14)	0.0282 (13)	0.0337 (14)	−0.0057 (11)	0.0044 (11)	−0.0067 (11)
Cd1	0.02621 (11)	0.03603 (12)	0.04251 (12)	−0.01089 (8)	0.00477 (8)	−0.00660 (10)
Cl1	0.0287 (3)	0.0578 (4)	0.0431 (4)	−0.0050 (3)	0.0039 (3)	0.0038 (3)
Cl2	0.0364 (4)	0.0389 (4)	0.0533 (4)	−0.0110 (3)	0.0141 (3)	−0.0121 (3)
N1	0.0256 (11)	0.0377 (13)	0.0396 (13)	−0.0070 (9)	0.0014 (10)	0.0005 (10)
N2	0.0336 (13)	0.0449 (14)	0.0375 (13)	−0.0157 (11)	0.0039 (10)	0.0001 (11)
N3	0.0265 (12)	0.0396 (13)	0.0380 (13)	−0.0141 (10)	0.0039 (10)	−0.0025 (10)
N4	0.0288 (12)	0.0355 (13)	0.0330 (12)	−0.0111 (10)	0.0039 (9)	−0.0015 (10)

### Geometric parameters (Å, °)

**Table:**

C1—N1	1.389 (3)	C9—C14	1.403 (3)
C1—C2	1.393 (4)	C10—C11	1.373 (4)
C1—C6	1.399 (4)	C10—H10	0.9300
C2—C3	1.372 (4)	C11—C12	1.403 (4)
C2—H2	0.9300	C11—H11	0.9300
C3—C4	1.389 (5)	C12—C13	1.370 (4)
C3—H3	0.9300	C12—H12	0.9300
C4—C5	1.371 (4)	C13—C14	1.393 (4)
C4—H4	0.9300	C13—H13	0.9300
C5—C6	1.386 (4)	C14—N4	1.389 (3)
C5—H5	0.9300	Cd1—N4	2.305 (2)
C6—N2	1.381 (3)	Cd1—N1	2.338 (2)
C7—N1	1.317 (3)	Cd1—Cl2	2.4602 (8)
C7—N2	1.341 (3)	Cd1—Cl1	2.5725 (10)
C7—C8	1.450 (4)	Cd1—Cl1^i^	2.5903 (10)
C8—N4	1.327 (3)	Cl1—Cd1^i^	2.5903 (10)
C8—N3	1.348 (3)	N2—H15	0.8600
C9—N3	1.385 (3)	N3—H16	0.8600
C9—C10	1.387 (4)		
			
N1—C1—C2	131.0 (3)	C13—C12—C11	121.7 (3)
N1—C1—C6	108.9 (2)	C13—C12—H12	119.1
C2—C1—C6	120.1 (3)	C11—C12—H12	119.1
C3—C2—C1	117.6 (3)	C12—C13—C14	117.4 (3)
C3—C2—H2	121.2	C12—C13—H13	121.3
C1—C2—H2	121.2	C14—C13—H13	121.3
C2—C3—C4	121.8 (3)	N4—C14—C13	130.9 (2)
C2—C3—H3	119.1	N4—C14—C9	109.0 (2)
C4—C3—H3	119.1	C13—C14—C9	120.2 (3)
C5—C4—C3	121.3 (3)	N4—Cd1—N1	73.49 (8)
C5—C4—H4	119.3	N4—Cd1—Cl2	118.63 (6)
C3—C4—H4	119.3	N1—Cd1—Cl2	102.95 (6)
C4—C5—C6	117.4 (3)	N4—Cd1—Cl1	144.04 (6)
C4—C5—H5	121.3	N1—Cd1—Cl1	93.11 (6)
C6—C5—H5	121.3	Cl2—Cd1—Cl1	96.65 (3)
N2—C6—C5	132.8 (3)	N4—Cd1—Cl1^i^	91.81 (6)
N2—C6—C1	105.5 (2)	N1—Cd1—Cl1^i^	154.49 (6)
C5—C6—C1	121.7 (3)	Cl2—Cd1—Cl1^i^	102.39 (3)
N1—C7—N2	113.1 (2)	Cl1—Cd1—Cl1^i^	86.78 (3)
N1—C7—C8	119.9 (2)	Cd1—Cl1—Cd1^i^	93.22 (3)
N2—C7—C8	127.0 (2)	C7—N1—C1	105.3 (2)
N4—C8—N3	112.6 (2)	C7—N1—Cd1	112.71 (17)
N4—C8—C7	120.4 (2)	C1—N1—Cd1	141.87 (18)
N3—C8—C7	127.1 (2)	C7—N2—C6	107.1 (2)
N3—C9—C10	131.9 (2)	C7—N2—H15	126.4
N3—C9—C14	105.5 (2)	C6—N2—H15	126.4
C10—C9—C14	122.5 (3)	C8—N3—C9	107.4 (2)
C11—C10—C9	116.2 (3)	C8—N3—H16	126.3
C11—C10—H10	121.9	C9—N3—H16	126.3
C9—C10—H10	121.9	C8—N4—C14	105.6 (2)
C10—C11—C12	122.0 (3)	C8—N4—Cd1	112.97 (16)
C10—C11—H11	119.0	C14—N4—Cd1	140.52 (16)
C12—C11—H11	119.0		
			
N1—C1—C2—C3	179.2 (3)	C6—C1—N1—C7	1.1 (3)
C6—C1—C2—C3	−0.3 (5)	C2—C1—N1—Cd1	−2.8 (5)
C1—C2—C3—C4	1.3 (5)	C6—C1—N1—Cd1	176.7 (2)
C2—C3—C4—C5	−0.8 (5)	N4—Cd1—N1—C7	−4.14 (18)
C3—C4—C5—C6	−0.8 (5)	Cl2—Cd1—N1—C7	112.26 (18)
C4—C5—C6—N2	−178.4 (3)	Cl1—Cd1—N1—C7	−150.18 (18)
C4—C5—C6—C1	1.9 (5)	Cl1^i^—Cd1—N1—C7	−61.1 (2)
N1—C1—C6—N2	−0.7 (3)	N4—Cd1—N1—C1	−179.5 (3)
C2—C1—C6—N2	178.8 (3)	Cl2—Cd1—N1—C1	−63.1 (3)
N1—C1—C6—C5	179.1 (3)	Cl1—Cd1—N1—C1	34.5 (3)
C2—C1—C6—C5	−1.3 (4)	Cl1^i^—Cd1—N1—C1	123.5 (3)
N1—C7—C8—N4	5.0 (4)	N1—C7—N2—C6	0.7 (3)
N2—C7—C8—N4	−175.5 (3)	C8—C7—N2—C6	−178.9 (3)
N1—C7—C8—N3	−175.0 (3)	C5—C6—N2—C7	−179.7 (3)
N2—C7—C8—N3	4.6 (5)	C1—C6—N2—C7	0.0 (3)
N3—C9—C10—C11	178.9 (3)	N4—C8—N3—C9	−0.1 (3)
C14—C9—C10—C11	0.0 (4)	C7—C8—N3—C9	179.8 (3)
C9—C10—C11—C12	0.1 (5)	C10—C9—N3—C8	−178.9 (3)
C10—C11—C12—C13	−0.1 (5)	C14—C9—N3—C8	0.1 (3)
C11—C12—C13—C14	−0.2 (4)	N3—C8—N4—C14	0.1 (3)
C12—C13—C14—N4	−178.9 (3)	C7—C8—N4—C14	−179.9 (2)
C12—C13—C14—C9	0.4 (4)	N3—C8—N4—Cd1	171.44 (17)
N3—C9—C14—N4	0.0 (3)	C7—C8—N4—Cd1	−8.5 (3)
C10—C9—C14—N4	179.1 (2)	C13—C14—N4—C8	179.3 (3)
N3—C9—C14—C13	−179.5 (2)	C9—C14—N4—C8	0.0 (3)
C10—C9—C14—C13	−0.3 (4)	C13—C14—N4—Cd1	11.9 (5)
N4—Cd1—Cl1—Cd1^i^	88.65 (9)	C9—C14—N4—Cd1	−167.4 (2)
N1—Cd1—Cl1—Cd1^i^	154.46 (6)	N1—Cd1—N4—C8	6.56 (18)
Cl2—Cd1—Cl1—Cd1^i^	−102.11 (3)	Cl2—Cd1—N4—C8	−89.44 (19)
Cl1^i^—Cd1—Cl1—Cd1^i^	0.0	Cl1—Cd1—N4—C8	78.4 (2)
N2—C7—N1—C1	−1.1 (3)	Cl1^i^—Cd1—N4—C8	165.38 (18)
C8—C7—N1—C1	178.5 (2)	N1—Cd1—N4—C14	173.4 (3)
N2—C7—N1—Cd1	−178.16 (18)	Cl2—Cd1—N4—C14	77.4 (3)
C8—C7—N1—Cd1	1.4 (3)	Cl1—Cd1—N4—C14	−114.8 (3)
C2—C1—N1—C7	−178.4 (3)	Cl1^i^—Cd1—N4—C14	−27.8 (3)

Symmetry codes: (i) −*x*+2, −*y*+2, −*z*+1.

### Hydrogen-bond geometry (Å, °)

**Table:**

*D*—H···*A*	*D*—H	H···*A*	*D*···*A*	*D*—H···*A*
N3—H16···Cl2^ii^	0.86	2.39	3.221 (2)	163

Symmetry codes: (ii) *x*−1/2, *y*−1/2, *z*.
